# Baroreflex activation therapy reduces frequency and duration of hypertension-related hospitalizations in patients with resistant hypertension

**DOI:** 10.1007/s10286-020-00670-9

**Published:** 2020-02-12

**Authors:** Marcel Halbach, David Grothaus, Fabian Hoffmann, Navid Madershahian, Kathrin Kuhr, Hannes Reuter

**Affiliations:** 1 Department of Internal Medicine III, University of Cologne, Kerpener Str. 62, 50937 Cologne, Germany; 2 Department of Cardiac Surgery, University of Cologne, Cologne, Germany; 3 Institute of Medical Statistics and Computational Biology, University of Cologne, Cologne, Germany; 4 Evangelisches Klinikum Köln-Weyertal, Weyertal 76, 50931 Cologne, Germany

**Keywords:** Baroreflex, Hypertension, Hospitalization, Medical device, Resistant hypertension

## Abstract

**Purpose:**

Baroreflex activation therapy (BAT) has been shown to lower blood pressure in patients with resistant hypertension. The purpose of this study was to analyze whether this translates into a reduction of more relevant clinical endpoints.

**Methods:**

Patients with resistant hypertension were treated with the second-generation BAT system. Records on hospitalization (dates of admission and discharge, main diagnosis) were obtained from medical insurance companies.

**Results:**

Records on hospitalization were available for a period of 1 year before BAT in two patients and 2 years in 22 patients. The total number of hospitalizations per patient was 3.3 ± 3.5/year before BAT and 2.2 ± 2.7/year after BAT (*p* = 0.03). Hospitalizations related to hypertension were significantly decreased from 1.5 ± 1.6/year before BAT to 0.5 ± 0.9/year after BAT (*p* < 0.01). The cumulative duration of hypertension-related hospital stays was significantly reduced from 8.0 ± 8.7 days/year before BAT to 1.8 ± 4.8 days/year after BAT (*p* < 0.01). Office cuff blood pressure was 183 ± 27 mmHg over 102 ± 17 mmHg under 6.6 ± 2.0 antihypertensive drugs before BAT and 157 ± 32 mmHg over 91 ± 20 mmHg (both *p* < 0.01) under 5.9 ± 1.9 antihypertensive drugs (*p* = 0.09 for number of drugs) at latest follow-up. Daytime ambulatory blood pressure was 164 ± 21 mmHg over 91 ± 14 mmHg before BAT and 153 ± 21 mmHg (*p* = 0.03) over 89 ± 15 mmHg (*p* = 0.56) at latest follow-up. Heart rate was 75 ± 16 bpm before BAT and 72 ± 12 bpm at latest follow-up (*p* = 0.35).

**Conclusions:**

Rate and duration of hypertension-related hospitalizations in patients with severe resistant hypertension were lowered after BAT. Whether the response is mediated through improvements in blood pressure control requires further studies.

**Electronic supplementary material:**

The online version of this article (10.1007/s10286-020-00670-9) contains supplementary material, which is available to authorized users.

## Introduction

Resistant hypertension is a major determinant of cardiovascular complications [1–4]. Therapeutic options for patients with resistant hypertension include baroreflex activation therapy (BAT) [5, 6], i.e. electrical stimulation of baroreceptors located at the carotid artery. For BAT, a relevant reduction of systolic blood pressure was found in a number of trials. The only randomized, placebo-controlled trial was conducted with the first-generation system [5]. A total of 265 patients were randomized 2:1 to immediate or delayed (after 6 months) BAT activation. Systolic blood pressure was lower in activated vs. nonactivated patients after 6 months, but this was not significant due to an unexpected blood pressure drop in the control group. Significantly more patients with an active device reached goal blood pressure after 6 months, and sustained efficacy was demonstrated after 12 months, when both groups were activated. Six-year follow-up data showed a sustained reduction in blood pressure and argue against usually shorter-lasting placebo or Hawthorne effects [7].

The currently available second-generation device has been tested in smaller trials without a control group. The first trial found a 26-mmHg reduction of systolic blood pressure in 30 patients. An indirect comparison of first- and second-generation devices confirmed a similar blood pressure lowering effect [6]. A number of small trials further support the efficacy of the second-generation device [8–12]. In addition to reducing blood pressure, several trials found positive effects of BAT on other surrogate parameters, including glomerular filtration rate [13], proteinuria [14] and arterial stiffness [15]. De- and reactivation of BAT led to a significant acute blood pressure response [10]. In a randomized crossover study with 4 weeks of de- and reactivation, ambulatory blood pressure monitoring (ABPM) and office cuff measurements revealed a 10-mmHg difference between deactivated and activated patients, while home blood pressure measurements did not show a benefit [8].

Reduction of blood pressure prevented cardiovascular endpoints in randomized trials with single- or dual-drug therapy [16]. There have been no randomized trials supporting an effect of ≥ 3 drugs or any interventional approach on relevant cardiovascular endpoints; however, trials were designed to demonstrate a reduction of blood pressure as a surrogate marker of cardiovascular endpoints. Hospitalization is a highly relevant endpoint, since it is associated with morbidity and mortality [17, 18] as well as healthcare costs [19]. It is an indicator of net benefit, since it is driven by efficacy as well as safety events. Due to its frequent occurrence, it is a suitable endpoint to be studied in small populations.

To assess the effect of BAT not only on blood pressure but on a more clinically relevant endpoint, we analyzed the number and duration of hospitalizations in patients with resistant hypertension before and after treatment with BAT.

## Methods

This study is a retrospective analysis of hospitalization data obtained from medical insurance companies and blood pressure recordings performed during routine clinical follow-up of BAT patients.

### Device

BAT was applied by the second-generation system (Barostim neo™, CVRx, Minneapolis, MN, USA) as described before [6, 20]. The system consisted of a small dot-shaped electrode (platinum-iridium disc coated with iridium oxide attached to a circular insulative backer) connected to a pulse generator. The electrode was sutured onto the arterial wall in the carotid sinus region. The lead was tunneled subcutaneously and connected to a pulse generator implanted in the subclavicular region. Devices were activated approximately 2 weeks after implantation, and the intensity of stimulation was up-titrated during sequential follow-up visits until goal blood pressure was achieved or local side effects of stimulation occurred.

### Protocol

Thirty consecutive patients with resistant hypertension, i.e. blood pressure ≥ 140/90 mmHg despite antihypertensive therapy with ≥ 3 drugs from different classes including one diuretic and after exclusion of secondary causes of hypertension, treated with BAT at the University of Cologne, Germany, between February 2011 and June 2015 were included in this analysis. BAT is approved in Europe for treatment of resistant hypertension.

The primary endpoint of the study was overall and hypertension-related hospitalizations before and after BAT. Secondary endpoints included blood pressure obtained by office cuff measurements and ABPM. Based on our clinical experience with BAT and previous trials, we hypothesized that BAT was associated with a significant reduction in hypertension-related hospitalizations and a significant reduction in blood pressure in our cohort. Data were analyzed retrospectively. The analysis was considered as exploratory, and no effect size was defined as clinically relevant before data analysis was performed.

Medical insurance companies of the patients were asked to provide the date and duration of hospitalizations and main diagnosis according to ICD-10 classification. Hospital visits were only counted as a hospitalization if the patient spent at least one night in the hospital; i.e. visits to the out-patient or emergency department with discharge on the same day were not assessed. Data on hospitalizations during 2 years before device implantation were collected as baseline (“before BAT”); hospitalization rates earlier than 2 years before implantation were considered not representative as baseline due to estimated disease progression. Follow-up after device implantation (“after BAT”) varied according to the implantation date of patients; collection of follow-up data ended on July 15, 2016. Number and duration of hospitalizations were annualized to account for different durations of follow-up; only data of fully completed years were included in the analysis. Scheduled hospitalization for implantation of the BAT device was not counted as a hospitalization, while hospitalizations after device implantation but before activation were counted as “after BAT,” to assess all adverse events related to the procedure. Hospitalizations were classified as hypertension-related if ICD-10 codes I.10–I.13 were documented as the main diagnosis (I.15 [secondary hypertension] was excluded). Hospitalizations related to cardiovascular diseases included ICD-10 codes I.00–I.99 (excluding I.10–I.13).

Blood pressure measurements were part of the routine follow-up on BAT at the University of Cologne. Patients had monthly follow-up visits until 6 months and were examined in 3- or 6-month intervals thereafter. Office cuff blood pressure measurements were performed by trained physicians or nurses on patients sitting quietly with an appropriately sized cuff placed on the same arm each time, using an automated BPtru device (VSM Medtech, Vancouver, BC, Canada) set to calculate the mean of five measurements taken in 2-min intervals. ABPM was performed by our center or the general physician or cardiologist of the patient using devices of different manufacturers. Since blood pressure values were collected retrospectively and did not follow a predefined schedule, we did not perform blood pressure analyses at specific time-points after BAT activation, but focussed the analysis on the latest follow-up available for each patient.

### Statistics

Statistical analyses were performed using IBM SPSS Statistics Version 24 (IBM, Armonk, NY, USA). Variables of interest were described using mean values ± standard deviation (SD) or frequencies and percentages. Since sample size was small and we were not confident to have normally distributed data, we used nonparametric tests. To compare baseline and follow-up values, we used Wilcoxon sign rank tests for continuous variables and McNemar tests for categorial variables (paired samples). To compare subgroups of interest, we used Mann–Whitney *U* tests for continuous variables and Fisher’s exact tests for categorial variables (independent samples). The correlation between baseline systolic blood pressure and the reduction of hospitalizations was evaluated by the Spearman rank correlation coefficient. All reported *p* values are two-sided, and *p* values < 0.05 were considered statistically significant. As the analyses were regarded as explorative, we did not adjust for multiple testing.

Graphs were created using SigmaPlot 8.0 (IBM, Armonk, NY, USA) and Powerpoint 2016 (Microsoft, Redmond, WA, USA).

## Results

### Patient characteristics and device settings

Data from 30 patients were included in the analysis. Baseline characteristics of the patients are presented in Table 1. Stimulation settings at baseline and follow-up are shown in Supplemental Tables S1 and S2.

**Table 1:** Baseline characteristics

Variable	*n*	Mean ± SD or *n* (%)
Female	30	16 (53%)
Age, years	30	56 ± 14
Weight, kg	26	89 ± 19
Body mass index, kg/m^2^	26	31 ± 6
Blood pressure, mmHg		
Systolic	27	183 ± 27
Diastolic	27	102 ± 17
Heart rate, beats/min	23	81 ± 17
Number of antihypertensives	30	6.6 ± 2
RAS-I	30	29 (97%)
MRA	30	18 (60%)
β-blocker	30	29 (97%)
CCB	30	25 (83%)
Loop diuretic	30	16 (53%)
Thiazide	30	24 (80%)
Sympatholytic	30	25 (83%)
Vasodilator	30	9 (30%)
History of sleep apnea	30	4 (13%)
History of chronic kidney disease	30	15 (50%)
History of diabetes	30	8 (27%)
History of hyperlipidemia	30	23 (77%)
History of smoking	30	11 (37%)
History of CAD/PAD	30	8 (27%)
History of heart failure	30	17 (57%)
History of renal denervation	30	16 (53%)

*SD* standard deviation, *n* number, *CAD* coronary artery disease, *CCB* calcium channel blocker, *MRA* mineralocorticoid antagonist, *PAD* peripheral arterial disease, *RAS-I* inhibitor of the renin angiotensin system

### Office cuff blood pressure

**Fig. 1:**
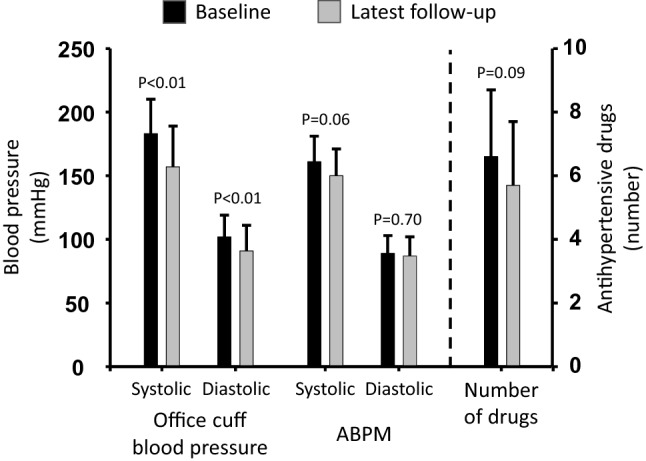
Blood pressure and antihypertensive drugs. Systolic and diastolic office cuff blood pressure decreased significantly from baseline to the latest follow-up in patients with resistant hypertension treated with BAT (left). ABPM decreased numerically (middle). The number of antihypertensive drugs was significantly lower at the latest follow-up than before BAT (right). *ABPM* ambulatory blood pressure monitoring

Office cuff recordings at baseline and latest follow-up were available for 27 patients. The latest follow-up was 652 ± 352 days after activation. Before BAT, office cuff blood pressure was 183 ± 27 mmHg over 102 ± 17 mmHg (Table 2, Fig. 1). At latest follow-up after BAT activation, office cuff blood pressure was reduced to 157 ± 32 mmHg over 91 ± 20 mmHg (both *p* < 0.01). Blood pressure after implantation but before device activation was available for 10 patients and amounted to 176 ± 34 mmHg over 103 ± 22 mmHg (*p* = 0.31 and 0.68 vs. baseline, both *p* = 0.05 vs. latest follow-up).

**Table 2:** Office cuff blood pressure

Characteristic	Baseline	Latest follow-up	Difference	*p* value
Systolic blood pressure				
No. of patients	27	27	27	
Mean ± SD, mmHg	183 ± 27	157 ± 32	−26 ± 31	
Median [IQR], mmHg	173 [162; 210]	147 [138; 171]	−23 [−37; −10]	< 0.01
≥ 180 mmHg, no. (%)	13 (48)	5 (19)		
< 140 mmHg, no. (%)	0 (0)	8 (29.6)		
Diastolic blood pressure				
No. of patients	27	27	27	
Mean ± SD, mmHg	102 ± 17	91 ± 20	−11 ± 17	
Median [IQR], mmHg	100 [90; 120]	87 [79; 100]	−6 [−19; −2]	< 0.01
≥ 110 mmHg, no. (%)	10 (37)	4 (15)		
< 90 mmHg, no. (%)	7 (26)	15 (56)		
Heart rate				
No. of patients	23	23	23	
Mean ± SD, bpm	81 ± 17	72 ± 14	−9 ± 21	
Median [IQR], bpm	79 [66; 89]	69 [60; 84]	−1 [−26; 7]	< 0.01

*p* values are from Wilcoxon signed rank tests

*SD* standard deviation, *IQR* interquartile range, *Bpm* beats per minute

### ABPM

ABPM recordings at baseline and latest follow-up were available for 23 patients. Latest follow-up was 517 ± 333 days after activation. Blood pressure assessed by 24 h ABPM was 161 ± 20 mmHg over 89 ± 14 mmHg at baseline and 150 ± 21 mmHg (*p* = 0.06) over 87 ± 15 mmHg (*p* = 0.70) at latest follow-up (Supplemental Table S3, Fig. 1). Changes in daytime but not in nighttime systolic blood pressure reached statistical significance (Supplemental Table S4).

The maximum blood pressure during ABPM did not change after BAT (*p* = 0.91 for systolic blood pressure, *p* = 0.59 for diastolic blood pressure; Supplemental Table S3). The percentage of hypertensive systolic blood pressure values during the 24-h monitoring period (daytime > 135/85 mmHg, nighttime > 120/75 mmHg) decreased significantly from 83 ± 18% before BAT to 70 ± 22% after BAT (*p* = 0.03). The percentage of patients with mean systolic blood pressure > 180 mmHg was numerically reduced from 23% before BAT to 9% after BAT.

Heart rate was 75 ± 16 bpm before BAT and 72 ± 12 bpm at latest follow-up (*p* = 0.35).

### Number and duration of hospitalizations

**Fig. 2:**
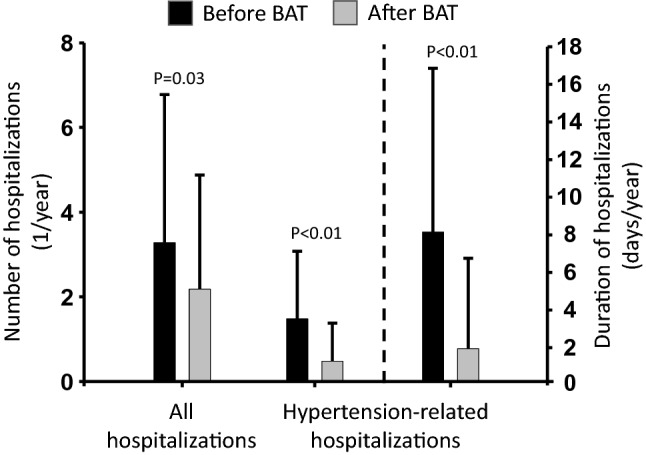
Rate and duration of hospitalizations before and after treatment with BAT. The number of all-cause hospitalizations was significantly reduced after BAT (left). This difference was solely driven by a significant drop in the number of hypertension-related hospitalizations (middle). The cumulative duration of hypertension-related hospitalizations was also reduced significantly after BAT (right)

Records on hospitalization were available for 24 patients, with a period of 2 years before BAT activation in 22 patients and 1 year before BAT activation in two patients. A list of all main diagnoses leading to hospitalization is included in the supplement (Supplemental Appendix). Follow-up after BAT activation was 1259 ± 498 days. The number of all-cause hospitalizations was 3.3 ± 3.5/year before BAT and was significantly reduced to 2.2 ± 2.7/year after BAT (*p* = 0.03, Table 3, Fig. 2). This difference was solely driven by a significant drop in hypertension-related hospitalizations from 1.5 ± 1.6/year to 0.5 ± 0.9/year (*p* < 0.01). Hospitalization rates related to cardiovascular diseases (except hypertension) or end-organ damage were not altered significantly (Table 3).

**Table 3:** Rate and duration of hospitalizations

Characteristic	Before BAT	After BAT	Difference	*p* value
All hospitalizations				
Hospitalizations/year	3.3 ± 3.5	2.2 ± 2.7	−1.1 ± 3.5	0.03
Hospitalizations related to cardiovascular events				
Hospitalizations/year	0.8 ± 1.7	0.6 ± 0.8	−0.2 ± 1.1	0.84
Days/year	4.4 ± 9.4	3.1 ± 4.3	−1.3 ± 6.0	0.57
Hospitalizations related to organ damage				
Hospitalizations/year	0.7 ± 1.5	0.6 ± 0.8	−0.1 ± 1.0	0.88
Days/year	3.4 ± 7.4	3.1 ± 4.2	−0.3 ± 5.0	0.92
Hospitalizations related to hypertension				
Hospitalizations/year	1.5 ± 1.6	0.5 ± 0.9	−1.0 ± 1.6	< 0.01
Days/year	8.0 ± 8.7	1.8 ± 4.8	−6.2 ± 9.8	< 0.01

Data were available for 24 patients. Values are displayed as mean ± standard deviation. *p* values are from Wilcoxon signed rank tests

*SD* standard deviation, *IQR* interquartile range, *Bpm* beats per minute

The cumulative duration of hypertension-related hospitalizations was significantly reduced from 8.0 ± 8.7 days/year before BAT to 1.8 ± 4.8 days/year after BAT (*p* < 0.01, Table 3, Fig. 2). The reduction of hypertension-related hospitalizations (both number and duration) was already observed in the first year after BAT activation and was preserved during the entire follow-up period (Supplemental Table S5).

The BAT device was replaced in nine patients during follow-up (once in six patients, twice in two patients and three times in one patient). First replacement was performed 2.4 ± 0.9 years after initial activation. Duration of hospitalization for replacement was 2.0 ± 1.3 days, when all cases are included in the analysis, and 1.6 ± 0.53 days when excluding one case involving perioperative stroke requiring a prolonged hospitalization period of 14 days.

### Subgroup analyses

Patients with a baseline systolic blood pressure above median (≥ 165 mmHg) showed a trend towards a higher rate and duration of hypertension-related hospitalizations before BAT (Supplemental Table S6). After BAT, the reduction of number as well as duration of hypertension-related hospitalizations was numerically higher in patients with baseline systolic blood pressure ≥ 165 mmHg, but these findings were not statistically significant. There was a small correlation between magnitude of baseline systolic blood pressure and the reduction of number and duration of hypertension-related hospitalizations; i.e. the higher systolic blood pressure was at baseline, the more pronounced was the reduction of hospitalizations (*r* = −0.453 for number of hospitalizations and *r* = −0.453 for duration). Subgroup analyses of patients with/without prior renal denervation (Supplemental Table S7), age above/below median (Supplemental Table S8) and male/female gender (Supplemental Table S9) revealed no significant differences between subgroups in the reduction of number or duration of hypertension-related hospitalizations after BAT.

### Number and classes of antihypertensive drugs

At baseline, the number of antihypertensive drugs was 6.6 ± 2.0. It was numerically reduced to 5.9 ± 1.9 drugs at latest follow-up (*p* = 0.09). The number of different drug classes was 5.8 ± 1.4 at baseline and 5.6 ± 1.6 at latest follow-up (*p* = 0.54). The percentage of patients taking a specific class of drugs did not change from baseline to the latest follow-up (all *p* > 0.05, Supplemental Table S10).

## Discussion

Here, we report a reduction of hypertension-related and all-cause hospitalizations after initiation of BAT in patients with resistant hypertension. In addition, a blood pressure lowering effect of BAT was confirmed.

One randomized, placebo-controlled trial with the first-generation device [5] and several noncontrolled trials with the second-generation device [6, 8–12], which is approved for treating resistant hypertension in Europe, have provided evidence that BAT reduces blood pressure in patients with resistant hypertension. These trials were focussed on the effect of BAT on blood pressure as a surrogate marker of cardiovascular endpoints, but none of the available trials assessed any cardiovascular endpoints or hospitalization data. Thus, the present study provides the first evidence of a potential reduction of hospitalization rate and duration after treatment with BAT, which is relevant due to the known association of hospitalization with morbidity and mortality [17, 18] as well as healthcare costs [19, 21].

To the best of our knowledge, other device-based therapies for resistant hypertension have not proven a beneficial effect on hospitalization. In the SYMPLICITY II trial [22], three patients with renal denervation and two control patients were admitted to the hospital for hypertensive crisis. In the SYMPLICITY III trial [23], hospitalization for new-onset heart failure or atrial fibrillation was observed in 4% of patients in the renal denervation group and 2.4% of patients in the sham procedure group (*p* value not reported). For medical treatment of hypertension, a beneficial effect on hospitalization rates has been demonstrated [24, 25]).

The rate of all-cause hospitalizations was reduced following BAT, but this difference was solely driven by a significant reduction of hypertension-related hospitalizations, while hospitalizations not related to hypertension were unaffected. This points to a potential causal link between BAT, the observed reduction in blood pressure and the positive effect on hospitalization. Interestingly, not only hospitalization rate was improved, but the duration of hospitalization was reduced in a relevant magnitude. This finding implies that in addition to the reduction in frequency of hypertensive crises requiring inpatient treatment, the severity and refractoriness to treatment may have improved after BAT.

Consistent with a significant reduction of office cuff blood pressure, daytime systolic blood pressure assessed by ABPM was significantly reduced after BAT. Nighttime and overall ABPM were numerically reduced, but this did not reach statistical significance. This lack of statistical significance is probably related to the high standard deviation and low number of patients, since significant reductions in nighttime and overall ABPM were described in other trials and were of similar magnitude as in our study [12, 14]. However, it cannot be excluded that BAT is less effective at night, which could be attributed to a lower sympathetic tone at night [26]. It is unlikely that reduction of blood pressure or hospitalization rate and duration were driven by changes in medication, since the number of antihypertensive drugs was numerically reduced after BAT and the prescribed classes of drugs were not altered in a relevant way.

Before data analysis was performed, no effect size was defined as clinically relevant. Few studies focussed on the effect of antihypertensive treatment on hospitalization, and definitions of hospitalization, underlying causes and statistical methods varied across studies; thus, interpretation and comparison of effect sizes is difficult. For instance, poor adherence to medication was associated with a hazard ratio of 1.47 for all-cause hospitalization as compared to good adherence [27]. Losartan reduced hospitalization for heart failure by 32% [28]. In view of these previous findings, the 33% reduction of the number of all-cause hospitalizations and the 66% reduction of the number of hypertension-related hospitalizations observed after BAT appear to be of relevance and roughly comparable to the effects of established therapies. The effect of BAT on blood pressure in the present trial (26 mmHg reduction of systolic office cuff blood pressure, 10 mmHg reduction of systolic blood pressure in ABPM) is equivalent to previous BAT trials [6, 8] and the effect of renal denervation in the treatment arm [29]. It is well documented that blood pressure reductions in this order of magnitude are associated with a highly relevant reduction of cardiovascular endpoints [2].

### Limitations

This study is subject to the limitations inherent in retrospective analyses and small patient numbers. However, our findings regarding blood pressure reduction are consistent with a number of previous trials, and it is well-known and plausible that such a significant reduction in blood pressure has a beneficial effect on hospitalization.

In addition, a placebo or Hawthorne effect is likely to have contributed to the observed blood pressure reduction and subsequent decrease in hospitalizations, especially in view of the invasive nature of BAT. The importance of such effects in interventional treatment of hypertension is underlined by the overestimation of therapeutic benefit in non-sham-controlled as compared to sham-controlled trials [23, 29]. However, the sustained reduction in blood pressure reported after 6 years of BAT [7] makes it unlikely that blood pressure reduction is exclusively driven by placebo or Hawthorne effects, since these effects usually last shorter.

Data on hospitalizations were provided from medical insurance companies, which collect dates and ICD-10-coded main diagnoses of hospitalizations from hospitals for reimbursement purposes. Therefore, coding errors, especially regarding the main diagnosis leading to hospitalization, cannot be excluded. It was not possible to verify duration and cause of hospital stays, since we did not have access to source data. Due to the high number of hospitalizations and the identical mode of data acquisition before and after BAT, this is unlikely to have influenced the overall results of the study.

Although we used an automated device for office cuff blood pressure assessments and routinely performed recordings in patients sitting quietly, there was no standardized protocol for office cuff measurements. ABPM measurements were even performed by different healthcare providers with devices from different manufacturers and did not follow a specified protocol. This may have contributed to the high standard deviation of blood pressure values and limits interpretation of data.

## Conclusion

We provide first evidence that the rate and duration of hospitalizations in patients with severe resistant hypertension may be reduced by BAT. This finding suggests that the known positive effect of BAT on blood pressure may potentially translate into a reduction of relevant clinical endpoints. Future studies involving larger patient numbers and addressing the effect of BAT on cardiovascular and cerebrovascular endpoints including mortality are needed and encouraged by the present findings. Moreover, the potential effect of BAT on hospitalizations may contribute to the cost-effectiveness of this therapy, which deserves further attention in future trials.

## Electronic supplementary material

Below is the link to the electronic supplementary material.

Supplementary file1 (DOCX 60 kb)

Supplementary file2 (DOCX 92 kb)
