# Correction to: Baroreflex activation therapy reduces frequency and duration of hypertension‑related hospitalizations in patients with resistant hypertension

**DOI:** 10.1007/s10286-021-00819-0

**Published:** 2021-07-23

**Authors:** Marcel Halbach, David Grothaus, Fabian Hoffmann, Navid Madershahian, Kathrin Kuhr, Hannes Reuter

**Affiliations:** 1 Department of Internal Medicine III, University of Cologne, Kerpener Str. 62, 50937 Cologne, Germany; 2 Department of Cardiac Surgery, University of Cologne, Cologne, Germany; 3 Institute of Medical Statistics and Computational Biology, University of Cologne, Cologne, Germany; 4 Evangelisches Klinikum Köln-Weyertal, Weyertal 76, 50931 Cologne, Germany

## Correction to: Clinical Autonomic Research (2020) 30:541–548 10.1007/s10286-020-00670-9

The article “Baroreflex activation therapy reduces frequency and duration of hypertension‑related hospitalizations in patients with resistant hypertension”, written by Marcel Halbach, David Grothaus, Fabian Hoffmann, Navid Madershahian, Kathrin Kuhr, and Hannes Reuter, was originally published electronically on the publisher’s internet portal on 12th February 2020 without open access. With the author(s)’ decision to opt for Open Choice, the copyright of the article changed on 7th July 2021 to © The Author(s) 2021 and the article is forthwith distributed under a Creative Commons Attribution 4.0 International License, which permits use, sharing, adaptation, distribution and reproduction in any medium or format, as long as you give appropriate credit to the original author(s) and the source, provide a link to the Creative Commons licence, and indicate if changes were made. The images or other third party material in this article is included in the article’s Creative Commons licence, unless indicated otherwise in a credit line to the material. If material is not included in the article’s Creative Commons licence and your intended use is not permitted by statutory regulation or exceeds the permitted use, you will need to obtain permission directly from the copyright holder. To view a copy of this licence, visit http:// creat iveco mmons. org/ licen ses/ by/4.0. Open access funding enabled and organized by Projekt DEAL.

The original article has been corrected.

