# Supplementary material for: Baroreflex activation therapy reduces frequency and duration of hypertension-related hospitalizations in patients with resistant hypertension
Source: Clin Auton Res. 2020 Feb 12;30(6):541–8. doi: 10.1007/s10286-020-00670-9 (PMC8302539; doi:10.1007/s10286-020-00670-9)
Supplement: Supplementary file 1 — Supplementary file1 (DOCX 60 kb) [file 10286_2020_670_MOESM1_ESM.docx]

**Full title:** Baroreflex activation therapy reduces frequency and duration of hypertension-related hospitalizations in patients with resistant hypertension

**Journal: Clinical Autonomic Research**

**Authors:** *Marcel HALBACH^1^, MD, *David GROTHAUS^1^, MD, Fabian HOFFMANN^2^, MD; Navid MADERSHAHIAN^3^, MD, Kathrin KUHR^4^, MSc, Hannes REUTER^5^, MD

*contributed equally

**Affiliations:**

^1^Department of Internal Medicine III, University of Cologne, Kerpener Str. 62, 50937 Cologne, Germany, This author takes responsibility for all aspects of the reliability and freedom from bias of the data presented and their discussed interpretation.

^2^Department of Internal Medicine III, University of Cologne, Germany, Contribution to this work: Collection of data

^3^Department of Cardiac Surgery, University of Cologne, Germany, Contribution to this work: Surgery, Collection of data

^4^Institute of Medical Statistics and Computational Biology, University of Cologne, Germany, Contribution to this work: Statistical analysis

^5^Department of Internal Medicine III, University of Cologne; Evangelisches Klinikum Köln-Weyertal, Weyertal 76, 50931 Cologne, Germany, This author takes responsibility for all aspects of the reliability and freedom from bias of the data presented and their discussed interpretation

**Corresponding author:** Marcel Halbach**;** Email: marcel.halbach@uk-koeln.de

**Supplemental Material**

**Supplemental Table S1. Statistical analysis of stimulation parameters**

| Characteristic | Baseline | Latest  Follow-Up | Maximum | Minimum |
| --- | --- | --- | --- | --- |
| Pulsewidth |  |  |  |  |
| No. of patients | 21 | 21 | 21 | 21 |
| Mean ± SD – µs | 115±107 | 167±158 | 333±171 | 102±142 |
| Median [IQR] – µs | 78 [55;125] | 125 [45;235] | 281 [141;500] | 63 [16;125] |
| Amplitude |  |  |  |  |
| No. of patients | 20 | 21 | 21 | 21 |
| Mean ± SD – mA | 6.2±2.2 | 6.8±3.4 | 8.7±4.3 | 3.6±2.0 |
| Median [IQR] – mA | 6.1 [5.0;7.0] | 6.2 [4.8;8.4] | 8.2 [6.4;10.4] | 4.0 [1.7;4.9] |
| Frequency |  |  |  |  |
| No. of patients | 21 | 21 | 21 | 21 |
| Mean ± SD – Hz | 52±23 | 62±17 | 74±17 | 41±17 |
| Median [IQR] – Hz | 40 [40;80] | 60 [50;80] | 80 [65;80] | 40 [25;55] |

Abbreviations: SD: Standard deviation; IQR: Interquartile range

**Supplemental Table S2. Stimulation parameters of individual patients.** Stimulation was active 24 h per day, i.e. deactivation of the therapy during a specific time frame of the day or night was not applied. In the minority of patients, a burst was transiently programmed, which is not included in the table for reasons of clarity.

| ID | PW  BL  in µs | Amp.  BL  in mA | FRQ  BL  in Hz | PW  LT  in µs | Amp.  LT  in mA | FRQ  LT  in Hz | PW  Max.  in µs | Amp. Max.  in mA | FRQ Max.  in Hz | PW  Min.  in µs | Amp.  Min.  in mA | FRQ  Min.  in Hz |
| --- | --- | --- | --- | --- | --- | --- | --- | --- | --- | --- | --- | --- |
| 1 | 234 | 5 | 40 | 250 | 5.2 | 50 | 250 | 9.6 | 100 | 63 | 4 | 30 |
| 2 | 156 | 7 | 20 | 220 | 6.2 | 40 | 281 | 7.4 | 65 | 125 | 6 | 20 |
| 3 | 250 | 4.8 | 40 | 205 | 5.2 | 60 | 281 | 6.8 | 80 | 205 | 4.8 | 40 |
| 4 | 16 | 6.4 | 20 | 31 | 7 | 50 | 125 | 8.2 | 80 | 16 | 5 | 20 |
| 5 | 47 | 6 | 40 | 65 | 7.4 | 80 | 141 | 7.4 | 80 | 16 | 4.6 | 20 |
| 6 | 31 | 6.2 | 20 | 125 | 6 | 80 | 500 | 9.6 | 80 | 31 | 1.6 | 20 |
| 7 | 125 | 7 | 40 | 500 | 2.4 | 80 | 500 | 7 | 80 | 31 | 2.4 | 40 |
| 8 | 78 | 6 | 80 | 500 | 1.6 | 80 | 500 | 1.6 | 80 | 500 | 1.6 | 80 |
| 9 | 94 | 8.6 | 80 | 500 | 2.6 | 50 | 500 | 2.8 | 80 | 500 | 2 | 50 |
| 10 | 78 | 8 | 80 | 140 | 9.4 | 60 | 155 | 9 | 80 | 125 | 8 | 60 |
| 11 | 78 | 7.8 | 80 | 65 | 4 | 80 | 500 | 4.4 | 80 | 65 | 1.4 | 60 |
| 12 | 78 | 6.2 | 80 | 65 | 10.4 | 65 | 500 | 10.4 | 80 | 63 | 1.8 | 65 |
| 13 | 125 | 5 | 80 | 45 | 8.2 | 100 | 500 | 10.4 | 100 | 15 | 1.6 | 65 |
| 14 | 500 | 1 | 80 | 15 | 6 | 40 | 500 | 11.2 | 80 | 15 | 1 | 40 |
| 15 | 63 | n/a | 60 | 45 | 13.5 | 50 | 125 | 5.2 | 80 | 16 | 1.8 | 40 |
| 16 | 125 | 4 | 60 | 45 | 15 | 80 | 500 | 14.5 | 80 | 30 | 4 | 40 |
| 17 | 31 | 12 | 60 | 30 | 7 | 60 | 125 | 19.5 | 65 | 15 | 2.8 | 40 |
| 18 | 125 | 6 | 40 | 280 | 6.8 | 40 | 500 | 17 | 40 | 125 | 6 | 40 |
| 19 | 65 | 4 | 20 | 125 | 5.2 | 40 | 250 | 6.8 | 40 | 65 | 4 | 20 |
| 20 | 30 | 6.2 | 40 | 110 | 8.6 | 60 | 110 | 8.6 | 60 | 30 | 6.2 | 40 |
| 21 | 95 | 6 | 40 | 155 | 4.4 | 60 | 140 | 6 | 40 | 95 | 4 | 40 |

Abbreviations: ID: Patient identification number; PW: Pulsewidth; Amp.: Amplitude, FRQ: Frequency, BL: Baseline, LT: Latest follow-up, Max.: Maximum; Min.: Minimum, n/a: not available

**Supplemental Table S3. Ambulatory blood pressure**

| Characteristic | Baseline | Latest Follow-Up | Difference | P-value |
| --- | --- | --- | --- | --- |
| Systolic blood pressure |  |  |  |  |
| No. of patients | 22 | 22 | 22 |  |
| Mean ± SD – mmHg | 161±20 | 150±21 | -10±23 |  |
| Median [IQR] – mmHg | 156 [141;175] | 150 [137;157] | -11 [-20;4] | 0.06 |
| Maximal blood  pressure* |  |  |  |  |
| Mean ± SD – mmHg | 202±25 | 205±30 | 3±28 |  |
| Median [IQR] – mmHg | 196 [184;225] | 201 [177;231] | 4 [-17;24] | 0.91 |
| Hypertensive blood pressure values† |  |  |  |  |
| Mean ± SD – % | 83±18 | 70±22 | -14±20 |  |
| Median [IQR] – % | 90 [62;97] | 75 [58;79] | -13 {-27;3] | 0.03 |
| Mean systolic blood pressure ≥180 mmHg – no. (%) | 5 (23) | 2 (9) |  |  |
| Mean systolic blood pressure <140 mmHg – no. (%) | 3 (14) | 8 (36) |  |  |
| Diastolic blood pressure |  |  |  |  |
| No. of patients | 22 | 22 | 22 |  |
| Mean ± SD – mmHg | 89±14 | 87±15 | -2±16 |  |
| Median [IQR] – mmHg | 89 [81;98] | 86 [79;94] | -2 [-13;14] | 0.70 |
| Maximal blood  Pressure*, |  |  |  |  |
| Mean ± SD – mmHg | 130±23 | 134±27 | 4±32 |  |
| Median [IQR] – mmHg | 135 [112;146] | 130 [115;145] | -4 [-19;27] | 0.59 |
| Hypertensive blood pressure values† |  |  |  |  |
| Mean ± SD – % | 57±31 | 52±27 | -5±32 |  |
| Median [IQR] – % | 71 [27;81] | 48 [27;64] | 0 [-25;28] | 0.93 |
| Mean diastolic blood pressure ≥110 mmHg – no. (%) | 1 (5) | 1 (5) |  |  |
| Mean diastolic blood pressure <90 mmHg – no. (%) | 12 (55) | 14 (64) |  |  |
| Mean arterial pressure |  |  |  |  |
| No. of patients | 21 | 21 | 21 |  |
| Mean ± SD – mmHg | 121±15 | 113±18 | -8±19 |  |
| Median [IQR] – mmHg | 120 [109;132] | 107 [105;119] | -12 [-22;8] | 0.08 |
| Heart rate |  |  |  |  |
| No. of patients | 22 | 22 | 22 |  |
| Mean ± SD – bpm | 75±16 | 72±12 | -3±14 |  |
| Median [IQR] – bpm | 72 [66;79] | 71 [61;80] | -4 [-6;4] | 0.35 |

P-values are from Wilcoxon-sign rank test. *Data were available for 16 patients. †Data were available for 15 patients.

Abbreviations: SD: Standard deviation; IQR: Interquartile range; Bpm: Beats per minute.

**Supplemental Table S4. Ambulatory blood pressure – daytime and nighttime measurements.**

| Time | Characteristic | Baseline | Latest Follow-Up | Difference | P-value |
| --- | --- | --- | --- | --- | --- |
| **Daytime** | Systolic blood pressure |  |  |  |  |
|  | Mean ± SD – mmHg | 164 ± 21 | 153 ± 21 | -11 ± 23 |  |
|  | Median [IQR] – mmHg | 162 [145;178] | 150 [135;162] | -12 [-21;5] | 0.03 |
|  | Diastolic blood pressure |  |  |  |  |
|  | Mean ± SD – mmHg | 91 ± 14 | 89 ± 15 | -3 ± 16 |  |
|  | Median [IQR] – mmHg | 93 [80;102] | 88 [80;96] | -1 [-13;10] | 0.56 |
| **Nighttime** | Systolic blood pressure |  |  |  |  |
|  | Mean ± SD – mmHg | 155 ± 26 | 147 ± 22 | -8 ± 26 |  |
|  | Median [IQR] – mmHg | 148  [136;177] | 145  [130 - 161] | -5 [-22;15] | 0.27 |
|  | Diastolic blood pressure |  |  |  |  |
|  | Mean ± SD – mmHg | 85 ± 15 | 82 ± 14 | -3 ± 17 |  |
|  | Median [IQR] – mmHg | 84 [76;95] | 81 [73;91] | -2 [-17;11] | 0.45 |

P-values are from Wilcoxon-sign rank test**.** Data for daytime measurements were available for 23 patients. Data for nighttime measurements were available for 22 patients.

Abbreviations: SD: Standard deviation; IQR: Interquartile range.

**Supplemental Table S5. Rate and duration of hospitalizations by year before/after BAT**

|  | 2 years before BAT  (n=22) | 1 year before BAT (n=24) | 1st year after BAT (n=24) | 2nd year after BAT  (n=20) | 3rd year after BAT  (n=16) | 4th year after BAT (n=9) | 5th year after BAT (n=5) |
| --- | --- | --- | --- | --- | --- | --- | --- |
| All hospitalizations  (hosp./year) | 3.1±3.7 | 3.4±3.8 | 2.0±2.7 | 2.2± 2.6 | 2.7±3.7 | 3.8±4.0 | 2.0±2.9 |
| Hospitalizations related to cardio-vascular events (hosp./year) | 0.9±2.0 | 0.8±1.8 | 0.4±0.8 | 0.5±1.1 | 1.0±1.5 | 0.8±1.3 | 0.2±0.5 |
| Hospitalizations related to cardiovascular events  (days/year) | 4.0±10.1 | 4.7±10.3 | 1.6±3.5 | 2.0±4.6 | 6.1±9.2 | 2.6±5.3 | 1.6±3.6 |
| Hospitalizations related to organ damage  (hosp./year) | 0.9±2.0 | 0.5±1.4 | 0.5±0.7 | 0.7±1.0 | 0.8±1.5 | 0.8±1.3 | 0.2±0.5 |
| Hospitalizations related to organ damage  (days/year) | 4.0±10.1 | 2.7±6.3 | 1.8±3.1 | 3.3±5.5 | 4.6±8.9 | 2.6±5.3 | 1.6±3.6 |
| Hospitalizations related to hypertension (hosp./year) | 1.2±2.0 | 1.7±1.6 | 0.8±1.7 | 0.5±1.0 | 0.4±0.7 | 0.3±1.0 | 0.4±0.6 |
| Hospitalizations related to hypertension (days/year) | 4.9±8.3 | 10.3±11.6 | 2.3±5.9 | 0.9±2.4 | 1.4±3.6 | 4.7±14.0 | 1.4±2.0 |

Values are displayed as mean ± standard deviation.

Abbreviations: BAT: Baroreceptor activation therapy.

**Supplemental Table S6. Hypertension-related hospitalization rate and duration by baseline systolic blood pressure**

|  | Rate (hosp./year)  <165.4 mmHg  (n=10) | Rate (hosp./year)  ≥165.4 mmHg  (n=11) | Duration (days/year)  <165.4 mmHg  (n=10) | Duration (days/year)  ≥165.4 mmHg  (n=11) |
| --- | --- | --- | --- | --- |
|  |  |  |  |  |
| Before BAT | 1.1±1.6 | 2.0±1.7 | 6.6±9.8 | 8.5±7.1 |
| After BAT | 0.4±0.6 | 0.7±1.2 | 1.4±3.3 | 2.6±6.4 |
| Difference | -0.8±1.5 | -1.3±1.9 | -5.2±10.0 | -6±9.6 |
| P-value | 0.14 | | 0.18 | |

Values are displayed as mean ± standard deviation. P-values are from Mann-Whitney-U test comparing differences between before and after BAT values in two independent samples.

Abbreviations: BAT: Baroreceptor activation therapy.

**Supplemental Table S7. Hypertension-related hospitalization rate and duration by prior renal denervation**

|  | Rate (hosp./year)  without RDN  (n=11) | Rate (hosp./year)  with RDN  (n=13) | Duration (days/year)  without RDN  (n=11) | Duration (days/year)  with RDN  (n=13) |
| --- | --- | --- | --- | --- |
|  |  |  |  |  |
| Before BAT | 1.1±1.8 | 1.8±1.5 | 4.4±6.1 | 11.1±9.5 |
| After BAT | 0.3±0.6 | 0.6±1.1 | 1.3±3.1 | 2.2±6.0 |
| Difference | -0.8±1.6 | -1.2±1.6 | -3.1±6.3 | -8.9±11.6 |
| P-value | 0.15 | | 0.05 | |

Values are displayed as mean ± standard deviation. P-values are from Mann-Whitney-U test comparing differences between before and after BAT values in two independent samples.

Abbreviations: RDN: Renal denervation; BAT: Baroreceptor activation therapy.

**Supplemental Table S8. Hypertension-related hospitalization rate and duration by age**

|  | Rate (hosp./year)  <53 years  (n=11) | Rate (hosp./year)  >53 years  (n=13) | Duration (days/year)  <53 years  (n=11) | Duration (days/year)  >53 years  (n=13) |
| --- | --- | --- | --- | --- |
|  |  |  |  |  |
| Before BAT | 1.3±1.1 | 1.7±2.1 | 7.9±8.1 | 8.1±9.7 |
| After BAT | 0.6±1.2 | 0.3±0.4 | 2.8±6.4 | 0.5±0.7 |
| Difference | -0.7±1.4 | -1.4±1.8 | -5.1±10.4 | -7.6±9.3 |
| P-value | 0.62 | | 0.82 | |

Values are displayed as mean ± standard deviation. P-values are from Mann-Whitney-U test comparing differences between before and after BAT values in two independent samples.

Abbreviations: BAT: Baroreceptor activation therapy.

**Supplemental Table S9. Hypertension-related hospitalization rate and duration by gender**

|  | Rate (hosp./year)  women  (n=11) | Rate (hosp./year)  men  (n=13) | Duration (days/year)  women  (n=11) | Duration (days/year)  men  (n=13) |
| --- | --- | --- | --- | --- |
|  |  |  |  |  |
| Before BAT | 1.7±1.3 | 1.3±1.8 | 9.6±8.1 | 6.7±9.3 |
| After BAT | 0.7±1.2 | 0.2±0.4 | 3.4±6.9 | 0.5±0.7 |
| Difference | -1.0±1.7 | -1.1±1.6 | -6.3±11.3 | -6.2±8.9 |
| P-value | 0.58 | | 0.58 | |

Values are displayed as mean ± standard deviation. P-values are from Mann-Whitney-U test comparing differences between before and after BAT values in two independent samples.

Abbreviations: BAT: Baroreceptor activation therapy.

**Supplemental Table S10. Number of antihypertensive drugs and percentage of patients taking a specific class of drugs.**

|  | Baseline | Latest follow-up | P-value |
| --- | --- | --- | --- |
| Number of antihypertensives | 6.6±2.0 | 5.9±1.9 | 0.09 |
| Number of drug classes | 5.8±1.4 | 5.6±1.6 | 0.54 |
| RAS-I (%) | 97 | 97 |  |
| MRA (%) | 60 | 63 |  |
| β-blocker (%) | 97 | 93 |  |
| CCB (%) | 83 | 90 |  |
| Loop diuretic (%) | 53 | 67 |  |
| Thiazide (%) | 80 | 60 |  |
| Sympatholytic (%) | 83 | 60 |  |
| Vasodilator (%) | 30 | 27 |  |

Abbreviations: CCB: Calcium channel blocker; MRA: Mineralocorticoid antagonist; RAS-I: Inhibitor of the renin angiotensin system.
