# Supplementary material for: Baroreflex activation therapy reduces frequency and duration of hypertension-related hospitalizations in patients with resistant hypertension
Source: Clin Auton Res. 2020 Feb 12;30(6):541–8. doi: 10.1007/s10286-020-00670-9 (PMC8302539; doi:10.1007/s10286-020-00670-9)
Supplement: Supplementary file 2 — Supplementary file2 (DOCX 92 kb) [file 10286_2020_670_MOESM2_ESM.docx]

**Supplemental Appendix.** **List of ICD-10-coded main diagnoses leading to hospitalization.**

| Pat. ID | Diagnoses before BAT | | Diagnoses after BAT | | | | |
| --- | --- | --- | --- | --- | --- | --- | --- |
|  | 2 years  before BAT | 1 years  before BAT | 1^st^ year  after BAT | 2^nd^ year  after BAT | 3^rd^ year  after BAT | 4^th^ year  after BAT | 5^th^ year  after BAT |
| 1 | - | I10.11 | - | Z45.9  Z45.9 | - | Z45.08 | - |
| 2 | - | - | - | Z45.80 | Z45.80 | - | - |
| 3 | G47.31  G47.31 | M23.33  M17.1  I71.03 | I50.13  Z45.88 | K42.9  Z45.88  J20.9 | M17.1  I71.4 | T85.1  T81.0  R06.0 | I50.13  I10.10 |
| 4 | X | I11.00  I10.11 | I10.10  T85.1 | - | - | E87.1  G25.81  F32.2  Z45.80  3 | K59.0  S30.0  S32.01  S32.01  K56.4  M48.06  F33.2 |
| 5 | I10.01  I10.01  I20.9  I10.01  I20.0  I20.0  I10.01  I10.01  I10.01 | I20.8  I10.01  I20.0  I10.01  T81.0  I50.13  I10.01  I20.0  I20.0  I20.0  I10.01  F41.1  F32.2  I10.01  I10.01  I20.0 | I10.01  K40.91  I10.01  I10.91  I25.13 | I20.0  I10.01  I20.8 | I63.5  I63.3  Z45.80  I20.0 | I21.0  L91.0  T82.5  I63.5  I20.9  I20.0 | I10.01 |
| 6 | - | I70.01  I10.10 | I10.10 | I61.8  I61.0 | - | I69.1 | X |
| 7 | I25.10  I84.1 | - | K42.9 | N18.5  T84.2  I10.10 | - | I25.10 | X |
| 8 | I20.0  I20.0  K92.0  I20.0  I20.0  M79.28  R55  I10.91  K21.0  I20.0  I20.0  I20.0  I20.0  I20.0  I10.01 | I20.0  R42  I10.01  R55  R55  I63.8  I10.01  I20.0  I20.0  I10.00  R07.3 | G45.82  I20.0  M17.1  K92.1  N20.1  I20.8  I10.01  I10.01 | M79.28  I10.10  K20  I63.4  I20.0  I20.0  I10.01  K80.21 | I20.0  I25.13  M25.56  I20.0  I20.0  I10.91  I50.11 | T84.0  Z45.80  R55  K29.6  R55 | X |
| 9 | I10.01 | I10.00  I10.10 | I10.11  I10.01  I10.91  I10.11  I10.11  I10.11  Z01.5  R07.2  I10.11  I20.0  I10.11 | J45.8  I10.11  I10.11  G45.12  E26.0  E26.0  I10.11  I10.11  D50.9 | R06.0  I10.11  J45.1  I50.13  I10.11  M54.18  R20.8  G83.1  D25.1  R52.1  G57.3  G83.1  G37.9 | K80.10  K80.20  I10.11  G62.88  I10.11  G57.3  G47.0  G04.9  I10.01  G61.8  J20.9  G61.8  G61.8 | X |
| 10 | J95.0  G40.1  G40.1  I10.01  I10.01  D14.1  G40.4 | I10.01  G40.6  I10.01  I10.10  I10.11 | I10.11 | G40.1 | Z45.80  G40.1 | X | X |
| 11 | X | I10.10  I11.91  I13.21  I50.14 | N20.1  N20.1  I11.00 | - | I50.13  I25.5  I48.0 | X | X |
| 12 | R51 | - | - | R06.0 | - | X | X |
| 13 | R15  I10.11  R15  R15 | R15  R15  T85.7  R15  T85.1  I10.11 | - | - | I10.11 | X | X |
| 14 | I67.4  N95.0  I10.91  I11.91  I10.11  I10.11  I11.90 | J44.03  J45.0  J45.9  I10.91  J44.12  I10.11 | M79.38 | Z45.80 | J44.09  I11.00  K80.10  R55  I45.5  I10.11  I44.2  G47.31 | X | X |
| 15 | - | - | I80.28  I20.0  I70.1  I72,4 | I20.0  I20.0  G40.3  I20.0 | - | X | X |
| 16 | I48.10 | I10.10 | - | - | Z45.80  I26.9 | X | X |
| 17 | T84.5  T84.5 | - | - | J20.9  K10.28 | X | X | X |
| 18 | I10.11 | I10.11  I10.11 | R26.8  N17.9  R55 | - | X | X | X |
| 19 | I10.91  I10.91  E11.41  I10.91  I10.91  I10.11  I10.91 | I10.91  I10.91  I10.91 | N17.9 | I10.91  T82.5  I10.11  E87.1 | X | X | X |
| 20 | I10.00 | F33.2 | - | - | X | X | X |
| 21 | I10.01 | I10.11  K42.9  M76.6 | G47.31 | X | X | X | X |
| 22 | M51.2  I63.3 | M19.08  I10.91 | K50.1 | X | X | X | X |
| 23 | O13  O11 | I10.91  I10.11  I10.01  I10.01 | - | X | X | X | X |
| 24 | I20.0  R55  I20.0 | I50.12  I20.8  I10.01  I49.5  I10.01  I10.91  I20.8 | I10.01  I50.13  I10.01 | X | X | X | X |

-: no hospitalization in the respective year; X: follow-up did not cover the complete year.
